# Exploring the Role of Active Assisted Living in the Continuum of Care for Older Adults: Thematic Analysis

**DOI:** 10.2196/40606

**Published:** 2023-05-22

**Authors:** Gaya Bin Noon, Thokozani Hanjahanja-Phiri, Harishree Dave, Laura Fadrique, Jennifer Teague, Plinio P Morita

**Affiliations:** 1 School of Public Health Sciences University of Waterloo Waterloo, ON Canada; 2 Communitech Waterloo, ON Canada; 3 Canadian Standards Association Toronto, ON Canada; 4 Research Institute for Aging University of Waterloo Waterloo, ON Canada; 5 Department of Systems Design Engineering University of Waterloo Waterloo, ON Canada; 6 Centre for Digital Therapeutics Techna Institute University Health Network Toronto, ON Canada; 7 Institute of Health Policy, Management, and Evaluation Dalla Lana School of Public Health University of Toronto Toronto, ON Canada

**Keywords:** ambient assisted living, active assisted living, AAL, internet of things, aging well, aging in place, older adults, geriatrics, standards, policies, health care

## Abstract

**Background:**

Active assisted living (AAL) refers to systems designed to improve the quality of life, aid in independence, and create healthier lifestyles for those who need assistance at any stage of their lives. As the population of older adults in Canada grows, there is a pressing need for nonintrusive, continuous, adaptable, and reliable health monitoring tools to support aging in place and reduce health care costs. AAL has great potential to support these efforts with the wide variety of solutions currently available; however, additional work is required to address the concerns of care recipients and their care providers with regard to the integration of AAL into care.

**Objective:**

This study aims to work closely with stakeholders to ensure that the recommendations for system-service integrations for AAL aligned with the needs and capacity of health care and allied health systems. To this end, an exploratory study was conducted to understand the perceptions of, and concerns with, AAL technology use.

**Methods:**

A total of 18 semistructured group interviews were conducted with stakeholders, with each group comprising several participants from the same organization. These participant groups were categorized into care organizations, technology development organizations, technology integration organizations, and potential care recipient or patient advocacy groups. The results of the interviews were coded using a thematic analysis to identify future steps and opportunities regarding AAL.

**Results:**

The participants discussed how the use of AAL systems may lead to improved support for care recipients through more comprehensive monitoring and alerting, greater confidence in aging in place, and increased care recipient empowerment and access to care. However, they also raised concerns regarding the management and monetization of data emerging from AAL systems as well as general accountability and liability. Finally, the participants discussed potential barriers to the use and implementation of AAL systems, especially addressing the question of whether AAL systems are even worth it considering the investment required and encroachment on privacy. Other barriers raised included issues with the institutional decision-making process and equity.

**Conclusions:**

Better definition of roles is needed in terms of who can access the data and who is responsible for acting on the gathered data. It is important for stakeholders to understand the trade-off between using AAL technologies in care settings and the costs of AAL technologies, including the loss of patient privacy and control. Finally, further work is needed to address the gaps, explore the equity in AAL access, and develop a data governance framework for AAL in the continuum of care.

## Introduction

### Background

Canada has a growing aging population, which has led to a pressing need for nonintrusive, continuous, and reliable health monitoring tools that can support aging in place [1-4] and reduce health care costs [4,5]. One of the biggest challenges in helping older adults continue to age in their own homes and communities is the increasing prevalence of chronic diseases as individuals continue to live longer [6]. Moreover, the COVID-19 pandemic has accelerated the need for remote monitoring tools that enable clinicians to support their patients from a distance with fewer clinic visits and hospital admissions [7,8].

Active assisted living (AAL) technologies can improve the quality of life, aid in independence, and create healthier lifestyles for those who need assistance at any stage of their lives. Three years ago, the demand for services and technologies that support telehealth, AAL, and internet of things (IoT) for health was not met, as many technologies were still in their infancy when the pandemic began [9]. Consequently, the pandemic incentivized the accelerated commercialization of products, and the market was flooded with products of lower quality [9]. This, in turn, increased the need for technology guidelines and an ecosystem capable of accommodating new technologies as they become available [10].

Other concerns arising from the rapid integration of AAL and IoT into care, such as the loss of care recipients’ privacy and control over their own information, have not been adequately addressed [11]. In addition, care recipients may fear the loss of independence owing to actions taken by caregivers and care providers based solely on AAL data or a growing dependence on the technology without making other considerations [2,9].

### Goal of This Study

The study was conducted in close collaboration with care providers and other stakeholders to ensure that the recommendations for system-service integrations aligned with the needs and capacity of the health care and allied health systems. This study also recognized that the individual needs of users extend beyond the home environment to include services and data collected at the community and city levels. Therefore, this was an exploratory study with the aim of understanding the opportunities for and challenges of integrating AAL technologies into the health system at the community level (eg, into the practice of paramedicine and other emergency services, pharmacies, allied health professional services, and medical clinics).

### Study Rationale

As a core goal of the AAL technology ecosystem is to promote independent living and improve the quality of life for vulnerable individuals, the authors not only considered the user’s home but also addressed the individual AAL requirements beyond the home environment, including services and data collected at the community and city levels. Unfortunately, AAL technologies are rarely integrated with external services, especially community health services [11-13]. The primary use of AAL in the continuum of care is to support integrated care [14], and successful integrated care depends on seamless transitions between care services and settings. Integrated care should also include coordinated care that offers access to services within a reasonable time frame, as well as effective treatment, self-care support, respect for care recipients’ preferences, and appropriate involvement of family members and other informal caregivers [14,15]. Some simple solutions identified in the literature for improving the continuity of AAL include accurate contact information for care providers and discharge information that is clear and tailored to the care recipient [16,17].

Over the years, attempts have been made to integrate IoT and AAL technologies into the homes of older adults; however, there are practical and financial considerations for developing and implementing these integrations [8,13,18]. Besides these considerations, there is a trade-off between health technology use and the right to privacy, but the incentive must be at least greater than the effort of learning how to use the technology and loss of one’s privacy because of sharing data with service providers [19-22]. Specifically, the advantages offered to older adults from adopting a health technology (ie, better support for independent living, reduced dependence on others, or the ability to navigate the physical environment of their home or care setting) must be perceived to be greater than the loss of privacy and perceived loss of control [11,22].

### Conceptual Framework

**Figure 1:**
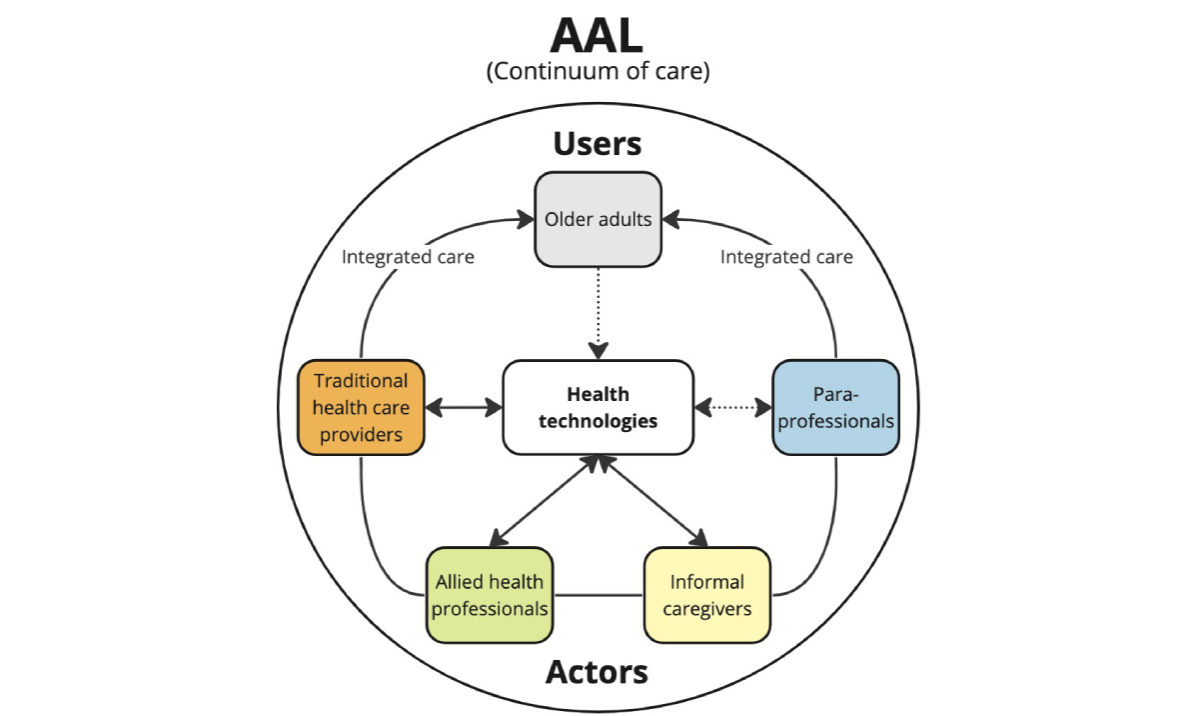
Existing landscape of active assisted living (AAL) technologies and their applications in the continuum of care.

In this conceptual framework for an AAL system, a *user* is a care recipient who becomes a *data participant* when their personal data are collected by different technologies. An *actor* refers to an entity that communicates and interacts in the system, including persons, technical components, software applications, systems, databases, and other bodies that play a role in the system. In an AAL system, an *agent* refers to any person interacting with the system, excluding the care recipient, or any person interacting with the care recipient along the continuum of care, including traditional health care provider users, allied health professionals, informal caregivers, and paraprofessionals. A visualization of the conceptual framework is presented in Figure 1.

*Traditional health care providers* are traditional in the sense that, in addition to being naturally associated with the term health care providers, past pilot projects that have sought to integrate data from AAL technologies into care typically first seek to include them. This group includes physicians, nurses, pharmacists, and social workers. Agents playing additional roles associated with the care of older adults, such as transition care coordinators or geriatric care managers, are included here if they perform these roles in the context of their work as a nurse or social worker. Allied health professionals refers to agents who work in the health care context and are paid through public funding but have not commonly been included in past pilot projects of integrated AAL systems. Agents in this group can still benefit from the data obtained from AAL systems (eg, data related to movement, room temperature, and use of tracked or radio-frequency identification–tagged items) if they are properly interpreted and targeted to their work. Examples of agents in this group include physical therapists, occupational therapists, patient navigators, community health workers, and personal support workers. Informal caregivers refers to agents who are not compensated for the care they provide and are involved in the continuum of care owing to their personal relationship with the care recipient. These agents are usually family members and close friends, and in some cases, they may act as the legal guardian or substitute decision maker if the care recipient has a high degree of impairment. Informal caregivers who live with the care recipient, such as a spouse or other cohabitants, are in a unique situation with regard to the AAL system because they also live in the smart living environment, so devices collecting data on the care recipient may also collect data on them. This raises the need to consider how the AAL system manages different needs, consents, and data sources.

*Paraprofessionals* are agents who directly impact the quality of life but are unlikely to directly interact with AAL technologies. Examples include agents hired for home maintenance, meal delivery, transportation, or housekeeping or volunteers who provide similar services. Finally, *health technologies* refers to any technology developed to prevent, diagnose, or treat medical conditions; promote health; provide rehabilitation; or organize health care delivery [23]. In the context of AAL, the goal of health technologies is to facilitate the wellness of and help maintain the independence of older adults.

## Methods

### Study Design

One round of 18 semistructured, small-group interviews was held with stakeholders involved in the care of older adults or development, manufacturing, or integration of care-related technologies between March and May 2022. This interview method was chosen because of its flexibility in obtaining targeted and unique perspectives of different stakeholders and understanding the interactions between different staff members in the same organization (where applicable) [24]. A semistructured interview guide with 16 questions was developed (Multimedia Appendix 1). An expert in the field of user-centered design and human factors methods provided guidance on the formulations of the questions for the interview.

In the interviews, the agents were asked for details about their experiences with the use of technology in the care of older adults; the interviews were guided by a data governance framework that was intentionally broad (ie, not AAL specific). Participants were recruited from the following four groups:

1. Care organizations: stakeholders working for organizations directly involved in care delivery (eg, retirement communities, long-term care homes, and community care organizations)
2. Technology developers: stakeholders working for companies or groups involved in the development or manufacturing of AAL technologies
3. Technology integrators: stakeholders working for companies or groups involved in the integration of AAL technologies into care
4. Reception: potential care recipients and patient advocates representing the interests of older adults

In total, >40 stakeholders from Canada and the United States were invited to participate in the interviews. Some of the stakeholders contacted also forwarded the study’s information to other groups that they felt would be interested in participating. An informational letter with a description of the project objectives was included in the invitation. After potential participants read the informational letter and confirmed their interest in participating, they received an informed consent letter to be signed before the interview appointment. In cases where the participant was not able to submit a signed consent letter before the interview, they were asked to verbally provide consent to participate in the study on the day of the interview, which was recorded and stored separately from the recording of the interview itself.

A total of 18 interviews were held comprising 1 to 3 participants per organization, depending on participant availability. The interviews with reception participants were held individually, as they were not representing an organization. Of the 18 interviews, 6 (33%) were held with care organizations, 4 (22%) with technology developers, 3 (17%) with technology integrators, and 5 (28%) with reception. The participants were assigned letters and numbers to deidentify them, ranging from P1 to P25.

The interviews were conducted over Zoom (Zoom Video Communications, Inc) and began with a brief presentation to contextualize the project. Following the presentation, questions were posed using a semistructured interview guide, which allowed for the questions to be adapted to each participant’s context. A basic version of the interview guide was shared with the participants before the interview to give them time to consider the questions. Whenever possible, the interviews were conducted with groups of participants who worked in different positions within the same organization to gather diverse perspectives. Following their interview, the participants received a feedback letter thanking them for their participation, reminding them of the purpose of the study, and providing them with details on confidentiality and ethics.

A thematic analysis of the interview transcripts was then conducted by summarizing the benefits, concerns, and barriers regarding the use of AAL data in the care of older adults and describing the current state of data flow in the context of AAL technologies. This method was chosen for its applicability when identifying topics within semistructured interviews [25,26]. After the first 4 interviews, open coding began, and a set of inductive codes was developed collaboratively with the members of the research team. These codes were revisited and revised iteratively as needed as new concepts emerged in the subsequent interviews. Coding was done using the NVivo software (QSR International) by GBN and HD. Six interviews were chosen at random to be coded by both GBN and HD to ensure consistency. Any discrepancies in coding were discussed between the 2 researchers, and conclusions were then applied to the remaining analysis. After this process, codes were grouped into themes and shared and discussed with the rest of the research team and an advisory panel of experts in older adult care for approval.

### Ethics Approval and Informed Consent

The procedure of this study and the semistructured interview guide, informed consent letter, and informational letter used in this study were reviewed and provided ethics approval by a University of Waterloo Research Ethics Committee (ORE# 43958). No risks were anticipated in the study, although the participants were warned that, given that they were being interviewed in a group setting, confidentiality could not be guaranteed from the other participants in the interview. No remuneration for participation was offered.

## Results

During the interviews, the participants described the benefits, concerns, and barriers that they perceived regarding the use of AAL data in the care of older adults. This section is divided among these 3 topics, with further subheadings to distinguish key insights provided by the participants.

### Benefits of AAL Systems

#### Monitoring and Alerting

The participants outlined several benefits that AAL can provide to the care of older adults at varying points in the continuum of care. The most consistently cited benefit was the potential of sensors and other monitoring technologies to predict and alert care providers and caregivers to incidents in which the care recipient is at risk, such as injury or illness. The system would detect deviations and abnormalities compared with the typical parameter metrics. For example, P11, who came from a technology development organization, described the value of their technology as follows:

Most senior people have some chronic condition or multiple chronic conditions, and with good electronic devices, we can collect data on a daily basis so that we can monitor their general health condition and identify problems early, right, before they become a big issue.

As described here, continuous monitoring can potentially detect when a care recipient is experiencing a decline by detecting deviations and abnormalities compared with their typical parameter metrics. In general, the participants described monitoring as taking 1 of 2 forms. The first was the tracking of regularly measured parameters to examine whether they have exhibited a recent change, with participants describing solutions such as smart thermometers and glucose monitoring (cited by participant P6) or blood pressure monitoring (cited by participant P8); the second was a more sophisticated option in which machine learning was used to identify the usual behaviors and patterns of the care recipient to establish a baseline for daily functioning, such as “monitoring activities of daily living” (cited by participant P6), “collecting data on (...) how they’re sleeping, when they’re getting up to go to the bathroom” (cited by participant P2), and “time spent (...) sitting, standing, lying” (cited by participant P11). The latter was described as preferable, as it allows care providers to be more proactive, with participant P4 explaining that “if someone has typically got normal morning routines (...) and you’re not seeing those routines and there’s alerts, we can go check on her” and several participants (participants P2, P8, P15, P21, P22, and P25) noting its potential to promote independence.

Health care providers can receive alerts of incidents, but more importantly, the continuous monitoring of data can accurately capture changes in the health of care recipients. These data can be used to adjust their care plan as needed. Such systems are particularly desirable, as they mitigate the burden of identifying and reporting relevant information about care recipients, especially about care recipients who cannot report on their own well-being because of cognitive or language difficulties. Participant P24 provided the example of a care recipient with a comorbidity of Parkinson disease and dementia who was experiencing issues with their movement (ie, freezing or hyperactivity) stemming from the dose and timing of medication delivery:

The problem with Parkinson’s is by the time you get to that degree of Parkinsonism, you have cognitive issues, and so trying to get people to remember how they were at some time during the day, it’s hard.

The use of AAL would allow for the circumvention of this issue, providing a record of when symptoms occurred that a care provider can then cross-reference against a medication schedule.

#### Confidence in Aging in Place

Another potential benefit described by the interview participants is a greater confidence in aging in place for both care recipients and their various caregivers. Two participants (P7, who came from a care organization, and P15, a technology integrator) remarked on the potential of technological supports to prevent “premature” moves into assisted care settings, allowing the older adult to be in their preferred space. Beyond the safety and convenience that AAL technologies provide, they can also offer caregivers peace of mind.

P16 (technology integrator) described how caregivers can benefit from AAL technologies:

For a family member or caregiver, being able to actually see the information around daily activities or movement (...) I see that playing important role in elder care so that they can better understand (...) what’s happening with their family member, because oftentimes what we’ve been finding (...) the individual older adult living in their home doesn’t want to share everything that’s happening, they don’t want to tell them about if they had a fall or if they’ve been feeling ill and staying in bed much longer than usual.

This was presented as an opportunity to reduce the burden of reporting among care recipients, as they may not want to share information that will worry their loved ones or make them appear unwell. By contrast, if all is well, the caregiver may feel more comfortable with not intervening and instead allowing the care recipient to continue aging in place.

#### Empowerment and Access

Some participants described how the use of AAL could contribute to greater access and empowerment with regard to care. Rather than going to a different care setting for assessment and adjustments, care recipients can get themselves assessed and their treatment adjusted without traveling and on their own schedule. P10 (technology developer) described these advantages:

Technology allows people who are more vulnerable in many ways to be able to access really quality services within the comforts of their home or wherever they are and get more help in a more real time and sustainable manner. Over time, these interactions add up to better care, more empowered care, more informed care in the long run.

Care recipients could access information about their own well-being in a timely manner and, ideally, in a manner that would be compatible with their health literacy level.

Another benefit highlighted during the interviews was the potential of AAL to not only aid in the management of care but also enrich care recipients’ lives. The participants (P1 and P2, who came from a care organization, and P14 and P16, technology integrators) discussed technologies’ potential to provide entertainment or connection or otherwise help the care recipient work toward something they would be motivated for, in other words, “meeting their motivation” (cited by participant P14).

### Concerns Regarding the Use of AAL Systems in Care

#### Data Governance

In Ontario, Canada, compliance with the Personal Health Information Act is crucial for health data gathered by devices [27]. When asked about their concerns regarding the current use of data from AAL technologies in care, many participants raised two key questions: (1) who owns and stores the data? and (2) what are they doing with them? The participants from care organizations and technology developers described this question as often being the first question asked when a new technology was proposed for care recipient use. Participant P1 (care organization) expressed further concerns:

Do I really know how things are being regulated, how it’s being stored? Not until I ask about it, right? It’s still very much up to me, and I think that’s by design. I would love to see the conversation shift (to) how information is being stored, or even to servers, like, are the servers in Canada, are they somewhere else? Those types of things are important.

For devices that gather nonhealth data, regulations or guidelines for the use and protection of data are less clearly defined. Participant P18 (a technology integrator) suggested that these questions reflect care recipients’ and health care providers’ discomfort with using the cloud infrastructure:

I guess the analogy I can give is that people are used to knowing that their data is sitting on a hard drive in the hospital, for example. It is not being sent to some cloud server which is holding data from that hospital, potentially next to someone’s Amazon shopping experience preferences or whatever else, right? And so, I think they there may be concern—I don’t have evidence for this exactly—but there may be some concern about mixed use of the information.

The same participant (P18) noted that users would sign away the rights to their data for their data to be used by algorithm developers and suggested that this might not be something a user would naturally agree to if they were aware of the terms:

We could frame it to them as like they’re accelerating the development of these algorithms by allowing us to use the data, but I’m wondering if there will be some that are hesitant in sort of signing off that data and realizing it’s not only a benefit to them specifically, for example.

However, this concern was not universally shared. Potential care recipients who were also interviewed (participants P21 and P22) did not express opposition to the idea when directly prompted, and a patient advocate (P23) offered a potential explanation:

One of the things that continually comes up (in conversations with policy groups is) people’s dissatisfaction with commercial uses of their health data. Generally speaking, people are totally happy for their deidentified health data to be used to improve health care services, improve public health, and to help other people (...) but when the data are used for commercial ends to make a company money then they just disapprove.

#### Accountability and Liability

As AAL systems continue to grow, more agents with different foci and concerns will be required. The participants raised concerns about this expansion, noting that when more agents are engaged with the care recipient, it would become more difficult to determine the true source of and, therefore, the solution for an incident. Participant P4, who came from a care organization, provided an example:

If in the unit, as an example, there’s a [proprietary emergency alert device], alerts for water leaks, alerts for smoke detectors. The smoke alarm went off, the toilet flooded, the door was opened. Which health authority does the smoke alarm fall under, the fire department or the infection control?

Furthermore, with alerting systems, there is the question of whether they could be held legally responsible for an incident resulting from a false positive or false negative they flagged. Participant P24 provided examples of how these systems might fail:

If [a technology used to identify aggressive behaviour] were to prompt a response inaccurately, if somebody was not engaging in an aggressive behavior, but a response was prompted against an aggressive behavior by long term care staff, that could cause a confrontation where previously there wouldn’t have been one (...) or having heart rate, blood pressure and weights displayed on the mirror in the bathroom for somebody with congestive heart failure, if there’s an inaccuracy in how that’s conveyed or if those are being built into an interpretive algorithm to indicate when to call a doctor, somebody places their trust in that, and it’s just displayed inaccurately for some reason, then that could cause a major medical event.

### Barriers to the Use and Implementation of AAL Systems

#### Value Trade-off

The participants raised many barriers to the use and implementation of AAL systems. The most heavily discussed of these was the perceived trade-off between the value of the technology and its cost and encroachment on care recipients’ privacy, as this calculation is key to agents’ willingness to invest and engage. Costs here can mean several things, with the most obvious meaning being financial expenses. AAL technologies are often expensive, and as participant P15 (a technology integrator) pointed out, there is little support available for interested users:

Technology is not provided in the form of a prescription. Therefore, there’s no pay model described, so who pays for it is always a big question. Although there could be a long list of benefits and validation as to why this is useful, whether for the health of the home or the health of the person, but the reimbursement model is not existent for it even with valid proof.

Similar concerns were expressed by the members of care organizations, with participant P3 noting that the situation may become further complicated for institutions procuring AAL rather than individual caregivers:

We talked about the cost factor, if it’s a cost that has to be absorbed by the client or its costs absorbed by the organization. Do you recoup the money from that? How does that work?

In addition, the participants expressed that an AAL system needed to be worth the time and effort involved in implementing and maintaining it. Participant P11 (a technology developer) referred to this as an “investment of time,” and participant P24 (a patient advocate) offered insight into what that time might be needed for:

Are providers given resources to adopt new technologies? Are they given extra slack time? Are they given education? Is there extra resource built into their day-to-day work so that they can take on issues that arise as they’re going through this adoption process?

Finally, regarding the privacy encroachment component of the trade-off, the participants noted that this affects the openness to the technology but emphasized the importance of understanding that the meaning of “privacy” may differ between a care recipient and others. Although the protection of users’ data is important, the focus that emerged in these interviews was more on a reluctance to be “spied on” or “nannied” by caregivers. Participant P17 (a technology integrator) explained this as follows:

[Privacy is] not where your data is going, that doesn't come up that often to be honest. It's about what people want. It's almost like if I want to get up and watch TV at 3am I don’t need an alert going to my son. I can do whatever I want (...) I have earned the right to do whatever I want.

#### Decision-making Process

Within an organizational context, the participants discussed issues related to the oversight and management of devices in an AAL system, as well as the expectations of management. Technology developers mentioned roadblocks such as the “long, slow decision chain” (participant P13) and “innovation-averse” nature of the Canadian health care system (participant P10). Furthermore, participant P11 discussed the difficult balancing act that technology developers must perform within the confines of health regulations:

In a long-term care facility, based on their current protocols, the nurse has to visit each room every two hours. If they couldn’t change that policy, that means even if they have new technology (...) they still have to visit this room every two hours. Then this will not save them any labor. (...) But then on the other hand, if the technology is really useful, it can reduce their workload, then the union will object because then some of them might fear they will lose their jobs.

Another aspect of management was the issue of care transitions with AAL systems. Participant P4 (care organization) described it as follows:

Some (devices) take a lot of time and pre-planning to set up into a room (...) but sometimes we get the calls that (a resident) can be released from the hospital, but they need these mechanisms in place, so we need to be able to pivot and set up a unit within hours and sometimes less to be able to accommodate that.

Agents working in care organizations must move swiftly to accommodate care recipients’ changing needs; however, the contexts in which they operate do not always allow for this. The participants expressed confusion regarding processes and procedures, which adds to their perception of effort.

#### Equity

Although equity was mentioned less frequently than other barriers, issues of equity cannot be ignored. The issue of cost potentially being prohibitive was already discussed; however, it must also be acknowledged that the affordability of devices is impacted by other factors. When defining “barriers,” a patient advocate (participant P23) posited the following:

[Barriers] implies that anything that interferes with use exists on the same plane, whereas (...) we live in a very high-level context that is a particular kind of capitalism, and that system incentivizes particular kinds of people to build particular kinds of technologies.

In some cases, the issues raised by the participants are not simply barriers to overcome but are in fact exclusionary roadblocks. Consideration of how to meet the needs of excluded groups is an important pursuit but may be outside the scope of this study.

## Discussion

### Overview

This study aimed to obtain recommendations from stakeholders regarding the best practices for system-service integrations for AAL. The recommendations were to align with the needs and capacity of health care and allied health systems. To this end, an exploratory study was conducted to understand stakeholders’ perceptions of and concerns with AAL technology use.

### Principal Findings

The participants discussed several potential benefits of the use of AAL systems, paying particular attention to the potential for more continuous monitoring. This may be especially valuable for the care of older adults with chronic conditions, and continuous monitoring could be used to detect when a care recipient is experiencing a decline and allow for more proactive care [28,29]. This relieves some of the burden of reporting from the care recipients themselves, meaning that they are not obligated to remember minute details or admit frailty [29,30]. That being said, any technologies brought into the home, for monitoring or other purposes, must be appropriate for the care recipient’s needs and consider the level of personal privacy and independence they wish to maintain.

Similar to the findings in the literature [13,20-22], one of the barriers discussed by some participants was whether the benefits of AAL systems justified the encroachment, real or perceived, on care recipients’ privacy, as well as the cost of the systems. The participants suggested that this may have been due to a lack of understanding of how these technologies work. Therefore, it would be beneficial to take the time to better inform care recipients of (1) the benefits of collecting data and (2) their own rights to choose and refuse the technology as desired, as well as to know which agents have access to their data. That being said, there was some disagreement regarding who that “privacy” is from, whether it be from external agents wanting access to the data, which is more common in the literature, or from informal care recipients with an interest in the care recipient’s daily activities.

Another part of this challenge is the perceived value of AAL systems when weighed against the financing, time, and resources necessary for their implementation because funding programs for home modifications vary between jurisdictions with no specific funding allocated to AAL. Therefore, care recipients and caregivers may struggle or be unable to afford these systems [31,32]. Even when the technologies are procured, it is still necessary to take time to learn how to use them and allow care providers to integrate them into their workflow and deal with any issues. The participants noted that the extra time required to incorporate these new practices often does not exist or is not accounted for during implementation of the AAL system.

Care facilities must contend with the fact that they do not necessarily have control or oversight over all devices in their facilities, as some devices might be brought in by care recipients and their families. There are advantages to this model, as it is often not feasible to bulk purchase devices at the facility level, and care recipients are more likely to agree with the use of a technology that they or their caregiver chose [33,34]. However, because the facility does not manage these devices, if there is an issue or a device failure of some sort (eg, a depleted battery), the facility would not be aware or be able to help, and there would be no way of integrating the data from these devices into the facility’s own alerting systems or record keeping unless a staff member were to maintain records of this data independently. In addition, AAL systems come with certain prerequisites that many homes and facilities lack. For example, Wi-Fi access is not guaranteed in facilities, and many facilities do not have a strong information technology team [35].

Another barrier described by the participants from care organizations was the difficulty in providing continuous care with AAL systems, such as when care recipients have moved from one care context to another. A dimension of this is the lack of interoperability between AAL systems and health information systems or among AAL systems abiding by the standards set by different manufacturers. If an entirely new system is required, the data from older devices may not be transferable. This lack of interoperability can add complexity, as care recipients’ data cannot follow them as technology evolves or as they move to different care settings [13,14,29]. Furthermore, the more comprehensive or detailed a solution is, the more time is required to set it up. However, when transitions need to happen very rapidly, this is not always feasible [35-38].

During procurement and implementation, managing the expectations and regulations of governing bodies, including unions, is particularly important. A recurring theme in interviews and literature was that health care providers were already overregulated to the point where regulations can get in the way of care [6,8,39,40]. The participants emphasized that if something is being introduced to the workflow, then governing bodies would need to consider what can be taken away to simplify procedures. Conversely, if the integration of the technology creates so much efficiency that the jobs of staff members could potentially become redundant, their union might object.

Governing bodies may raise valid concerns when integrating a new system that need to be addressed, such as compliance with regulations, budget concerns, and privacy concerns [13,39,41]. Two such concerns are technological capability and digital health literacy among both care recipients and their care partners [41]. Some participants expressed a concern that the eagerness to implement more technologically advanced and smart solutions has led to a skipping of “basic tech,” arguing that developers assume a level of competency that is not realistic. However, others argued that assuming that older adults cannot use technologies is agist and that they are highly capable of using technologies if the technologies are designed with their use in mind. Nevertheless, technologies are rarely designed for older adults’ use or able to accommodate the technological limitations of older adults with cognitive or dexterity issues [23,42].

There is also the question of how exactly care providers are expected to use the data from AAL systems. AAL systems generate a very high volume of data, leading to concerns among the interview participants about how these data can be used to improve care and coordination. Specific concerns included how alert fatigue can be prevented and how the data can be interpreted in a manner that is personal to the care of the recipient. Creating strategies for the interpretation of the data with the intended use in mind could then produce alerts for care providers that they could understand, trust, and act on to improve the health or well-being of a care recipient.

### Limitations

First, although this study used the term *barriers* for simplicity, the authors acknowledge the point made by participant P23 that this term does not account for the fact that the issues preventing AAL systems’ widespread use are multidimensional and sometimes exclusionary of marginalized groups. The equity aspects of access to AAL technologies in their current form include not being affordable for all, not being feasible for all, and often not being trusted by all [20,22,31]. Second, although the positions of a wide range of stakeholders were sought, it is not possible to conclude that all perspectives were represented. The study attempted to mitigate this by encouraging a debate between colleagues in the group interviews.

### Future Directions

This research points to a need for better clarity and role definition regarding the use of AAL systems in older adult care. Some pertinent issues include the lack of clarity regarding who can gain access to AAL data, how the data should be interpreted, which agent is responsible for action based on the collected data, and the trade-off of using AAL technologies in care settings. This may be addressed through further interdisciplinary research, including the development of a comprehensive data governance framework for AAL in the continuum of care.

In addition, future work should explore the external influences that guide the development of technologies at large. One such external influence is the fact that AAL development is often funded through a venture capitalist system that incentivizes technology companies to develop particular types of health technologies depending on what is most likely to be funded, rather than depending on evidence-informed needs [43-45]. Researchers should be cautious of assuming that the resources available to caregivers in a high-income, majority White, and socially connected semiurban community are also available to those elsewhere. If only the challenges of adoption and use faced by care recipients in this group are considered, then benefits will accrue only for them and enhance the health disparities faced by those who cannot afford AAL systems [31].

### Summary of the Findings and Conclusions

This exploratory study used a conceptual framework and interviews with participants to explore the needs and requirements for AAL systems and identify opportunities for standards in this area. The goal was to identify and understand the opportunities for and challenges of integrating these technologies into the health system at the community level (ie, into the practice of paramedicine and other emergency services, pharmacies, allied health professional services, and medical clinics). The study was conducted in close collaboration with care providers and other stakeholders to ensure that the recommendations for system-service integrations aligned with the needs and capacity of the health care and allied health systems.

The findings from our research have shown that although several potential benefits exist for the use of AAL systems within the continuum of care for older adults, additional work is needed to address concerns and barriers before these benefits can be fully realized. Much of the potential of AAL lies in its ability to support integrated care, meaning continuous and coordinated care that is quick, effective, and includes self-care support, respect for care recipients’ preferences, and appropriate involvement of family members and other informal caregivers [18,19]. AAL can improve care by facilitating better monitoring of care recipients’ health and empowering care recipients to pursue their health goals. However, to accomplish this, further work is necessary to define how data should be managed as AAL systems grow larger and more complex and more agents become involved, with an awareness of the perceptions of care recipients and their care partners as well as the equity issues inherent to AAL technology.

## Appendix

Multimedia Appendix 1 Semistructured interview guide.

## Abbreviations

AAL: active assisted living
IoT: internet of things

## Data Availability

The data sets generated or analyzed during this study are not publicly available because of confidentiality promised to the participants as part of the informed consent process but are available from the corresponding author upon reasonable request.
