# Supplementary material for: Exploring the Role of Active Assisted Living in the Continuum of Care for Older Adults: Thematic Analysis
Source: JMIR Aging. 2023 May 22;6:e40606. doi: 10.2196/40606 (PMC10242474; doi:10.2196/40606)
Supplement: Multimedia Appendix 1 [file aging_v6i1e40606_app1.docx]

**Semi-structured interview guide**

Questions were omitted or modified as appropriate for the type of organization or the participant’s background.

1. What kinds of health technology does your organization use to support care recipients and their health?
2. What are the barriers to using technology to provide care to care recipients?
3. How is the flow of information in retirement communities being handled today?
4. Who is involved in making decisions on behalf of care recipients regarding their data?
5. In what ways do you see health data from electronic devices playing an important role in elder care?
6. In your experience, how do you see health information being regulated?
7. What do you think of when I say “continuum of care”?
8. How do you see technology in elder care evolving in the next 5 years to support the continuum of care for seniors?
9. What types of living communities (e.g. LTCs, retirement, independent living) do you think have the most to gain from AAL? What communities are currently using it well?
10. What types of agents (e.g. doctors, pharmacists, nurses, non-clinical staff) have the most to gain from AAL? What agents are currently using it well?
11. What are some current regulatory challenges with the implementation or user acceptance of a new health technology in your organization?
12. What data security concerns or questions do you have when a new technology is proposed for use in your organization?
13. If you could wave a magic wand and change one thing in how information flows or is managed in senior care, what would it be?
14. We know the main goal is always to help the recipient of care. However, when it comes to health information, how can we bring the family into this process?
15. Who do you expect will be the trusted body or agency to oversee data governance in the AAL context?
16. Is there anything else you want to add/discuss?
